# The crime of surviving: Suicide recriminalization and mental health in Pakistan

**DOI:** 10.1371/journal.pmen.0000714

**Published:** 2026-08-31

**Authors:** Ahsan Mashhood, Murad Moosa Khan

**Affiliations:** 1 Department of Social Policy and Intervention, University of Oxford, Oxford, United Kingdom; 2 Department of Psychiatry, Aga Khan University, Karachi, Pakistan; PLOS: Public Library of Science, UNITED KINGDOM OF GREAT BRITAIN AND NORTHERN IRELAND

## Introduction: How survival became punishable again

On 18 May 2026, Pakistan’s Federal Shariat Court (FSC) ordered the restoration of Section 325 of the Pakistan Penal Code, which punishes attempted suicide with simple imprisonment for up to one year, a fine, or both [1, paras 7,33]. The Court held that the 2022 legislation removing Section 325 was contrary to the Holy Quran and Sunnah [1, para 33]. In legal terms, this is the reinstatement of a penal provision, while in public mental health terms, it recasts suicide survivors as criminals [1,2].

The law Pakistan has returned to is not new; it is part of a colonial legal inheritance that treated attempted suicide as an offence against the state [2,3]. For decades, this provision remained in the criminal code, even as global suicide prevention moved increasingly toward decriminalization and non-punitive responses to self-harm, e.g., the Decriminalize Suicide Worldwide Project [4,5].

In 2022, Pakistan briefly joined that shift and attempted suicide was decriminalized [6]. Pakistan nevertheless continued to face longstanding gaps in its suicide evidence base and mental health service capacity as research has routinely pointed out [7,8]. Decriminalization was, nonetheless, a step in the right direction and created active conversations on centering the survivor and thinking more deeply and critically about what leads individuals to attempt suicide [3,4].

However, that opening and optimism has narrowed and we are back to square one: once again revisiting the survivor as a liability. The 2026 ruling has often been framed as a question of religion: does Islam condemn suicide? [1] But this is not the only, or even the most useful, question. Many moral and religious traditions condemn suicide hence perhaps the more urgent question is what follows when the person is still alive: should the state respond to survival with punishment? [9] Should it make support easier to access, or should it allow a moment of acute distress to become the beginning of criminal liability? These are important questions because they reveal how a law that claims to protect life may, in practice, create additional barriers to help-seeking and care after a suicide attempt [4,10].

In this essay, we argue that Pakistan’s recriminalization of attempted suicide is a public mental health setback as it (re)turns a survivor into a possible accused and places emergency care in the shadow of criminal procedure. We examine this under three themes: 1) the legal logic through which punishment is framed as deterrence; 2) the unequal harms this produces for young people, women, and poorer families; and 3) the short and long-term consequences of recriminalization for mental health reform, suicide prevention, and the state’s duty of care. Because the FSC judgement was issued only in May 2026, this essay cannot present empirical estimates of the effects of the restored Section 325. Accordingly, we distinguish between four forms of evidence: findings from Pakistan during the pre-2022 criminalization regime; comparative evidence from jurisdictions where suicide attempts have remained criminalized; Pakistani evidence on stigma, gendered violence, poverty, and access to healthcare that identifies plausible mechanisms of harm; and prospective concerns about how these mechanisms may operate following the 2026 judgment. Where post-judgment evidence is not yet available, we describe these effects as risks or plausible consequences rather than established outcomes.

Furthermore, while our lens centers on Pakistan, we believe it has implications for comparable Global South contexts, especially Muslim-majority and/or Islamic Republics where suicide is either still a criminal offense, or only recently decriminalized and therefore still precarious.

## The legal logic of deterrence

The Federal Shariat Court’s restoration judgement of Section 325 sets out a theory of why attempted suicide should remain within the criminal law [1, paras 2–3,26–33]. In essence, the Court’s position is that because Islam treats life as sacred and suicide as prohibited, the state has the duty to retain punishment for an attempt to take one’s own life [1]. Furthermore, mental illness, in this framework, is not a reason to remove the offence altogether; it is, at most, a reason to exempt certain individuals from punishment on a case-by-case basis [1]. The petitions before the Court argued that Parliament’s 2022 decision to decriminalize attempted suicide was “contrary to the injunctions of Islam” [1, paras 2–3]. Human life, the petitioners argued, is a trust (amanaat) from God (Allah) and cannot be ended at one’s own discretion [1]. They also claimed that decriminalization could create wider legal problems, including leaving unpunished those who attempt suicide while of “sound mind,” and weakening the ability of the law to address abetment, encouragement, or facilitation of suicidal acts [1].

The Federation defended the 2022 amendment on public-health grounds [1]. Its position was that suicidal behavior often emerges in the context of depression, mental illness, or severe social and economic distress, and that punishment will worsen underlying crises [1,4]; that the survivor of attempted suicide requires treatment, counselling, and rehabilitation and not criminal sanctions by the state [1,4]. The Court rejected that argument, and a key part of its reasoning was that the parliamentary justification for decriminalization was too broad, particularly the suggestion that suicide attempts are “always” linked to mental illness or disorder [1].

The Court questioned whether every suicide attempt can be presumed to arise from mental illness and whether this logic might then be extended to other offences [1]. The Court’s conclusion was that mental illness may explain some cases but cannot justify repeal of the offence itself [1].

This led the Court to rely on existing criminal-law protections, especially Section 84 of the Penal Code, which exempts a person from liability if, at the time of the act, they were incapable, by reason of unsoundness of mind, of understanding the nature of the act or that it was wrong or contrary to law [1, paras 17–19; 2]. This is problematic as evidence from Pakistan shows that mental illness operates within a wider context of social stigma and disclosure, with consequences extending into family and marital relationships [11,12]. The Court also referred to the Code of Criminal Procedure provisions dealing with accused persons of unsound mind [1, paras 17–19; 13]. In effect, its position is that the law should keep attempted suicide as a crime for the public, while allowing mental incapacity to function as an exception [1,2]. This is the central legal logic of recriminalization: preserve punishment as a deterrent and treat mental illness through exemption rather than decriminalization [1]. The difficulty, however, is that this logic is far more coherent in legal theory than in clinical and public-health practice.

First, legal incapacity is a narrow threshold. Many people who attempt suicide may not meet the standard of ‘unsoundness of mind,’ even where depression, trauma, abuse, debt, shame, hopelessness, or family conflict are clearly central to the act; clinical vulnerability and legal incapacity are not the same thing [2,11,14]. A person may be in acute suicidal distress and in urgent need of care without being legally incapable of understanding what they are doing [2,11,14]. Second, the deterrent value of punishment in suicidal crisis is uncertain [4,15]. Criminal law assumes that the threat of sanction can shape behavior before the act occurs, yet many people in acute distress are not weighing the future possibility of imprisonment against their present suffering [4,12,16,17]. In Pakistan, some may know that suicide is religiously condemned, but may only encounter the legal implications of attempted suicide once they reach a hospital [11]. At that stage, the law may no longer deter the act; instead, it may deter disclosure, help-seeking, and meaningful documentation [4,10,11,18].

This is where, we argue, the judgment’s legal logic may become a public mental health problem [1,4]. A law meant to preserve life may, in practice, make it harder for survivors and families to seek care openly [4,10]. It also risks increasing concealment (which reproduces stigma), delaying treatment, and reducing the visibility of suicidal behavior within the health system [4,11,12,18]. Under Pakistan’s pre-2022 criminalization regime, clinicians reported concealment of self-harm and barriers to care linked to police and medico-legal involvement [10]. International evidence likewise does not show that criminalization reduces suicide rates [4,15]. The question, then, is not whether life should be protected; there is no disagreement there between us and the FSC [1]. It is whether criminalization creates the conditions through which life can be protected after a suicide attempt has occurred [4,10,16,17].

Furthermore, the religious prohibition of suicide does not, by itself, resolve the separate question of whether a person who survives an attempt must be criminally punished. Even if attempted suicide is considered within a ta‘zir framework, the relevant policy question remains whether criminal punishment of the survivor advances the purposes invoked to justify it, especially in relation to maqasid al-shariah and the preservation of life (hifz al-nafs), which the FSC has itself recognised as a primary objective of Shariah [1, paras 26,28,31]. Decriminalising the survivor need not imply legal indifference to coercion, abetment, exploitation, or facilitation of suicidal behaviour; such third-party conduct can be addressed separately without making the person who survives an attempt an accused person [1, paras 29–31]. Our argument is, accordingly, much narrower: the prohibition of suicide does not by itself determine that criminal punishment of the survivor is an appropriate means of protecting life.

## Who will suffer the most? Young people, women, and poor families

Criminal law is often written in general language and so it applies, in theory, to any person who attempts suicide. But laws are never experienced by abstract persons; they are experienced by people with different access to structural privilege, family power, mobility, money, social protection, and access to care. This is why the recriminalization of attempted suicide is also a problem of inequality, especially in a society as unequal as Pakistan. Hence the people most likely to be harmed by Section 325 are those whose suicidal distress is already influenced by dependence, gendered violence, financial precarity, and limited/no access to non-stigmatizing healthcare [3,7,10].

Young people are one such group. Evidence from Pakistan repeatedly suggests that suicidal behavior and self-harm are concentrated among younger people, often below the age of 30 or in early adulthood [14,19,20]. A recent Karachi-based study of self-harm found that 63% of cases were between 20 and 39 years of age and that interpersonal relationship issues were the most common reported reason for the attempt [20]. For a young person dealing with acute distress, especially one whose distress is linked to family conflict, academic pressure, relationship breakdown, violence, or shame, the hospital is already a difficult place to enter and so criminalization makes that entry even harder and leaves young people with little power in an already unequal society [3,7,10].

Like many South Asian countries, the family is a central institution in Pakistan [10]. Hence another layer of fear of punishment is the familial fear of exposure as a suicide attempt can quickly become a story about ‘izzat’, parental failure, sexual reputation, marriageability, religious sin, and/or police trouble [3,10]. In such a context, Section 325 gives families another reason to conceal the attempt, and/or delay disclosure, or reframe it as an “accident” rather than suicidal distress [10]. Hence given the Pakistani context, the law also changes the social calculations around whether a survivor should even be made legible/visible to the health system at all.

Women are likely to face a different, but equally serious, form of harm. In Pakistan, suicidal behavior cannot be understood only through the language of individual pathology; it is often entangled with domestic conflict, intimate partner violence, in-law dynamics, coercive marriages, restrictions on mobility, reproductive pressure, and everyday forms of gendered control [10,14,19]. Nationally representative data show that 34% of ever-married women in Pakistan have experienced physical, sexual, or emotional spousal violence, while 28% have experienced physical violence since age 15 and 6% have experienced sexual violence [21]. The same survey found that among women who had experienced physical or sexual violence, 56% had never sought help and had never told anyone [21]. This is the social world into which Section 325 has been restored.

Hence, for women, the danger of recriminalization is also that, as before, it may convert a crisis produced by violence into evidence of deviance: a woman who attempts suicide after abuse, marital conflict, coercion, or humiliation may already be blamed for bringing dishonor to the household [3,10,21]. If she survives, criminalization allows the meaning of the event to shift away from what happened to her and toward what she has allegedly done (e.g., harmed “izzat”). The survivor becomes the legal problem, and little attention is extended to the violence, neglect, abandonment, or coercive household that may have made life unbearable in the first place. In practice, then, this can strengthen the power of husbands, in-laws, or families who wish to silence women, especially young women. The threat of a police case, court involvement, public scandal, or damaged marriage prospects may become another instrument through which women are silenced and suppressed [10,21].

This is especially troubling because women’s (especially young women’s) access to care in Pakistan is already mediated by family authority and economic dependence. Many women cannot independently decide to seek healthcare, pay for treatment, travel to a hospital, or disclose violence to a doctor [21]. In the aftermath of a suicide attempt, the same people who control access to a woman’s care may also be the people implicated in the crisis. Criminalization therefore creates a perverse structure: the survivor may need protection from the household, but the medical-legal pathway may return her to that household under conditions of even greater shame and surveillance.

The burden may also fall heavily on poorer families, where the legal fiction of equal application collapses most clearly. Our ongoing conversations with frontline workers and medico-legal officers reveal that wealthier families can often purchase privacy, they may access private hospitals, consult private psychiatrists and overall avoid public medico-legal systems by negotiating with institutions through social capital, or reframe the event in ways that reduce exposure. Poorer families, on the other hand, may not have that luxury as they are more likely to rely on public emergency departments, which involve police-facing documentation. In other words, they are more visible to the state precisely because they have fewer alternatives to the state care system.

This is further compounded by the fact that healthcare in Pakistan (like other LMICs) is strongly shaped by out-of-pocket payments, and recent evidence shows that catastrophic health expenditure disproportionately affects poorer households [22,23]. A suicide attempt can bring emergency transport, hospital fees, medicines, follow-up care, psychiatric consultation, lost wages, and caregiving costs. Section 325 adds another possible layer: legal fees, informal payments (e.g., to the police), repeated visits to offices or courts, and the social costs of being known as a family with a “case.” For a daily-wage household, even one day spent navigating legal procedure may mean lost income, and for a poorer family, the fear of these costs may be high enough to avoid formal care altogether.

This is why deterrence is the wrong frame; it imagines a rational individual standing at some distance from the law, calmly weighing punishment against action. But the people most likely to be affected by Section 325 are not standing at equal distance from the law. Structural and socio-demographic factors (age, gender, socio-economic status) all interact together to shape experiences of who is exposed to police, paperwork and shame. Hence the law is unlikely to deter evenly, it may simply threaten selectively, especially those at the margins of the Pakistani society.

Recriminalization is then also an inequity multiplier: it may fall most heavily on those who lack power, money, privacy, or the ability to bend rules in the Pakistani context. An intersectional understanding of criminalization is useful here because these disadvantages can overlap, for example, age (young people), gender (women and gender minorities) and poverty may combine to make some survivors particularly vulnerable to the socio-institutional consequences of criminalization [3].

## What recriminalization makes precarious: the short and long term

At the time of writing this essay, psychiatric associations, mental health coalitions, civil society groups, and activist organizations in Pakistan are already mobilizing against the recriminalization of attempted suicide. Such resistance is important, and it may yet produce legal, political, or public-health pushback. But the fact that such mobilization is again necessary is itself revealing as it shows that legal reform in mental health is fragile: even when a punitive law is removed, it can return quickly, with little time for health systems, clinicians, survivors, or families to adjust. Hence the restoration of Section 325 also restores a deep uncertainty of mental health wins within the community.

In the short term, this uncertainty may divert already limited energy away from prevention and care as professional bodies, advocates, and mental health workers must now spend time defending a reform that should have been consolidated, rather than building the services that decriminalization was meant to enable: crisis response, survivor-centered care pathways, safe referral systems, public education, and better suicide surveillance. The immediate consequence that we are witnessing is legal confusion and potential policy displacement; e.g., attention is shifted from how to support people in suicidal distress to how to contest the state’s decision to punish them.

In the medium term, the ambiguity produced by recriminalization will likely be felt most sharply by those already exposed to discretionary power. When the law is unclear, or unevenly understood, or selectively enforced, it creates space for police pressure, family coercion, institutional avoidance, and bureaucratic intimidation. As argued in the previous section, such confusion is especially dangerous for young people, women, poor families, and other marginalized communities, whose access to privacy, legal protection, and non-stigmatizing care is already limited. For these groups the harm may arise simply from its availability: the possibility that a suicide attempt can become a police matter is enough to produce fear, silence, concealment, and delay.

In the longer term, Pakistan’s recriminalization demonstrates the precariousness of mental health rights when they are not anchored in durable systems. Indeed, it spotlights again how decriminalization alone was never sufficient [7,8]; it did not create a national suicide prevention strategy, expand mental health services, or repair emergency care. However, it created a direction of travel, and so recriminalization reverses that direction and signals that mental health protections are politically and legally fragile and can be unsettled at any moment.

The lesson extends beyond Pakistan. For countries that have recently decriminalized suicide (e.g., Malaysia and Ghana), especially those with similar colonial legal inheritances or religiously inflected legal systems, Pakistan’s experience shows that decriminalization must be defended, institutionalized, and translated into health infrastructure. Otherwise, reform remains vulnerable: a temporary legal opening rather than a permanent commitment to treating suicidal distress as a matter for care, not punishment.

## Conclusion: what should follow survival?

Given the urgency, we argue that decriminalisation should be accompanied by clear national protocols for emergency care: routine police involvement following self-harm should be restricted; confidentiality and medico-legal documentation standards should minimise unnecessary disclosure; survivors should receive psychosocial assessment, safe-discharge planning and appropriate follow-up; and pathways should be available for referral where domestic or gender-based violence is identified. Also, police, emergency clinicians and medico-legal staff should receive training and sensitization on non-punitive responses to suicidal crises, while improved surveillance and adequately funded community-based crisis services should form part of a national suicide-prevention strategy.

If repeal is delayed, several protections need not wait for legislative reform. Health authorities may immediately issue guidance limiting routine police referral, strengthening confidentiality, standardising psychosocial assessment and safe discharge, and ensuring that emergency treatment is never delayed because of potential criminal liability, especially in the context of health inequities we have outlined.

Furthermore, these reforms should also be developed with substantive lived-experience participation. For a start, this requires more than inviting survivors to share their stories after decisions have already been made: people with lived experience should have formal roles in the development and evaluation of legislation, hospital protocols, crisis services and suicide-prevention policy.

## References

[1] Federal Shariat Court of Pakistan. Judgement on prohibition of attempt to commit suicide: Shariat Petition No. 03/I of 2023, linked with Shariat Petition No. 01/I of 2025. Islamabad: Federal Shariat Court of Pakistan; 2026 [cited 2026 Aug 11]. Available from: https://www.federalshariatcourt.gov.pk/en/leading-judgements/

[2] Pakistan Penal Code, 1860 (Act XLV of 1860), ss 84, 325.

[3] Mashhood A, Saeed G, Sami F. Sociocultural perceptions of suicide in Pakistan: A systematic review & qualitative evidence synthesis. SSM - Mental Health. 2025;7:100433. doi: 10.1016/j.ssmmh.2025.100433

[4] World Health Organization. WHO policy brief on the health aspects of decriminalization of suicide and suicide attempts. Geneva: World Health Organization; 2023.

[5] Lifeline International. Decriminalise Suicide Worldwide [Internet]. [cited 2026 Aug 9]. Available from: https://www.suicide-decrim.network/

[6] Criminal Laws (Amendment) Act, 2022 (Act No. XXXVII of 2022).

[7] Shekhani SS, Perveen S, Hashmi D-E-S, Akbar K, Bachani S, Khan MM. Suicide and deliberate self-harm in Pakistan: a scoping review. BMC Psychiatry. 2018;18(1):44. doi: 10.1186/s12888-017-1586-6 29433468 PMC5809969

[8] World Health Organization. Mental Health Atlas 2020: member state profile—Pakistan. Geneva: World Health Organization; 2022.

[9] Cholbi M, Kious B. Suicide. In: Zalta EN, Nodelman U, editors. The Stanford Encyclopedia of Philosophy. Summer 2026 ed. Stanford (CA): Metaphysics Research Lab, Stanford University; 2026.

[10] Kiran T, Chaudhry N, Bee P, Tofique S, Farooque S, Qureshi A, et al. Clinicians’ Perspectives on Self-Harm in Pakistan: A Qualitative Study. Front Psychiatry. 2021;12:607549. doi: 10.3389/fpsyt.2021.607549 34093256 PMC8172994

[11] Naveed S, Qadir T, Afzaal T, Waqas A. Suicide and Its Legal Implications in Pakistan: A Literature Review. Cureus. 2017;9(9):e1665. doi: 10.7759/cureus.1665 29152422 PMC5677340

[12] Siddiq Shekhani S, Mashhood A, Hashmi D-E-S, Dossani-Lallany K, Asad N, Khan MM. Exploring effects of severe mental illnesses on marriages: A qualitative study from Karachi, Pakistan. PLOS Glob Public Health. 2025;5(12):e0005652. doi: 10.1371/journal.pgph.0005652 41433363 PMC12725543

[13] Code of Criminal Procedure, 1898, ch XXXIV, ss 464–75.

[14] Talha Z, Ahmad HS, Uzakova A, Shehzadi N, Shabbir S, Chaudary AS, et al. Evidence on Deliberate Self-Harm, Suicidal Ideation, and Suicides in Pakistan: A Scoping Review of Methods, Contributing Factors, and Associated Mental Disorders. Neuropsychobiology. 2026;1–25. doi: 10.1159/000552408 42118706 PMC13375204

[15] Wu KC-C, Cai Z, Chang Q, Chang S-S, Yip PSF, Chen Y-Y. Criminalisation of suicide and suicide rates: an ecological study of 171 countries in the world. BMJ Open. 2022;12(2):e049425. doi: 10.1136/bmjopen-2021-049425 35177441 PMC8860012

[16] Adinkrah M. Criminal prosecution of suicide attempt survivors in Ghana. Int J Offender Ther Comp Criminol. 2013;57(12):1477–97. doi: 10.1177/0306624X12456986 22923775

[17] Paternoster R. How much do we really know about criminal deterrence? J Crim Law Criminol. 2010;100(3):765–824.

[18] Wyllie JM, Robb KA, Sandford D, Etherson ME, Belkadi N, O’Connor RC. Suicide-related stigma and its relationship with help-seeking, mental health, suicidality and grief: scoping review. BJPsych Open. 2025;11(2):e60. doi: 10.1192/bjo.2024.857 40116563 PMC12001961

[19] Shahid M, Hyder AA. Deliberate self-harm and suicide: a review from Pakistan. Int J Inj Contr Saf Promot. 2008;15(4):233–41. doi: 10.1080/17457300802149811 19051086

[20] Tharani A, Farooq S, Lakhdir MPA, Talib U, Khan MM. Characteristics and patterns of individuals who have self-harmed: a retrospective descriptive study from Karachi, Pakistan. BMC Psychiatry. 2022;22(1):367. doi: 10.1186/s12888-022-04018-7 35641917 PMC9158237

[21] National Institute of Population Studies (NIPS) [Pakistan], ICF. Pakistan Demographic and Health Survey 2017–18. Islamabad, Pakistan, and Rockville (MD): NIPS and ICF; 2019.

[22] Iemmi V, Bantjes J, Coast E, Channer K, Leone T, McDaid D, et al. Suicide and poverty in low-income and middle-income countries: a systematic review. Lancet Psychiatry. 2016;3(8):774–83. doi: 10.1016/S2215-0366(16)30066-9 27475770

[23] Bashir S, Kishwar S, Nasir M, Ali S. Socioeconomic Inequalities in Out-of-Pocket and Catastrophic Health Expenditures in Pakistan. Int J Public Health. 2024;69:1607313. doi: 10.3389/ijph.2024.1607313 39600526 PMC11589653

